# The Impact of Posttransplant Lymphoproliferative Disease in High-risk Kidney Transplant Recipients: Benefits of Prevention

**DOI:** 10.1097/TXD.0000000000001792

**Published:** 2025-04-10

**Authors:** Bryce A. Kiberd, Christopher J.A. Daley

**Affiliations:** 1 Department of Medicine, Dalhousie University, Halifax, NS, Canada.; 2 Multi-Organ Transplant Program, Queen Elizabeth II Health Sciences Centre, Victoria Site, Halifax, NS, Canada.

## Abstract

**Background.:**

Posttransplant lymphoproliferative disease (PTLD) is increased in kidney transplant recipients who are Epstein-Barr virus (EBV) nonimmune (R^–^), particularly if the donor has prior EBV immunity (D^+^). PTLD is associated with very high mortality. The purpose of this study was to quantify the impact of PTLD on deceased donor EBV D^+^R^–^ kidney transplant recipients.

**Methods.:**

A Markov model was created to quantify remaining patient life years (LYs) and quality-adjusted LYs (QALYs) in EBV D^+^R^–^ recipients compared with EBV R^+^ recipients. Different ages at transplant, incidence of PTLD within the first year, potential impact of therapeutic treatments to reduce PTLD, and costs were examined in a sensitivity analysis.

**Results.:**

A baseline 40-y-old EBV D^+^R^–^ recipient is projected to live 21.18 LYs. If there is no PTLD, the recipient lives 21.37 LYs, but if PTLD develops in the first year, the projected life remaining LYs are only 15.03. Each high-risk 40-y-old EBV D^+^R^–^ recipient loses, on average, 0.192 LYs or 0.134 QALYs. LYs and QALYs gained with prevention depended on the effectiveness of the intervention, incidence of PTLD within the first year, and recipient age. Slightly fewer LYs are lost in younger recipients (age 10 y; 0.156 LF) and older recipients (age 60 y; 0.133 LY), likely due to lower case fatality rates and higher competing risks of death in the young and old, respectively. Strategies, such as rituximab, given at the time of transplant, could be cost-effective (<$50 000/QALY) if the reduction in PTLD was >50% and the cost of the intervention was <$3000.

**Conclusions.:**

PTLD has a significant impact on survival in high-risk kidney transplant recipients. Preventive strategies may be cost-effective but would depend on the degree of effectiveness, safety, and cost.

Posttransplant lymphoproliferative disease (PTLD) is increased 5- to 15-fold in Epstein-Barr virus (EBV)-naive kidney transplant recipients (R^–^) transplanted with EBV-positive donor kidneys (D^+^).^1,2^ PTLD is associated with a high mortality rate despite therapy.^1^ Currently, blood viral screening within the first year is recommended in EBV D^+^/R^–^ recipients, with a reduction in immunosuppression if the virus is detected in significant amounts.^3^ It is not clear how effective this screening is in reducing PTLD.

To reduce PTLD risk, EBV D^+^R^–^ transplantation could be avoided by transplanting EBV D^–^ donors into R^–^ recipients. Only 5%–6% of adult kidney transplant recipients are EBV naive, whereas the prevalence increases to >30% in pediatric recipients.^4^ More than 90% of donors are EBV^+^ by serology. Matching negative donors to negative recipients would reduce the availability of kidneys to EBV-naive recipients and could result in longer wait times. An alternative strategy is prevention with prophylaxis. Although a recent meta-analysis showed no statistically significant benefit, there was a trend for anti-CMV agents to reduce the risk.^5^

Rituximab is an important therapeutic option in PTLD. There is a growing body of evidence that rituximab prevents PTLD in hemopoietic cell transplantation (HCT).^6,7^ Rituximab given at the time of transplantation could be effective in solid organ transplantation, but this has not been studied rigorously.^8,9^ This approach is currently being examined (ClinicalTrials.gov ID NCT04989491) in kidney transplant recipients. However, the trial is expected to continue for 4 y or more. Some centers administer rituximab as preemptive therapy in patients who develop viremia that persists despite immunosuppression reduction.^7,10^

The 2 primary objectives of this study are to quantify the impact PTLD has on high-risk kidney transplant recipients and to assess the potential benefits of interventions that reduce the risk of developing PTLD. The secondary objectives were to examine the cost-effectiveness of rituximab and antiviral prophylaxis.

## MATERIALS AND METHODS

### Primary Analyses

#### Objective 1: Quantify the Impact of PTLD on a High-risk Kidney Transplant Recipient

A Markov model was created to examine remaining life years and quality-adjusted life years (QALYs) in a theoretical cohort of deceased donor kidney transplant recipients considered at high risk of PTLD (EBV D^+^/R^–^) compared with a cohort at normal-risk (EBV R^+^), where the risk of PTLD within the first year was considered negligible. After the first year, the risk of PTLD was assumed to be the same in both high-risk and normal-risk individuals.

**FIGURE 1.**
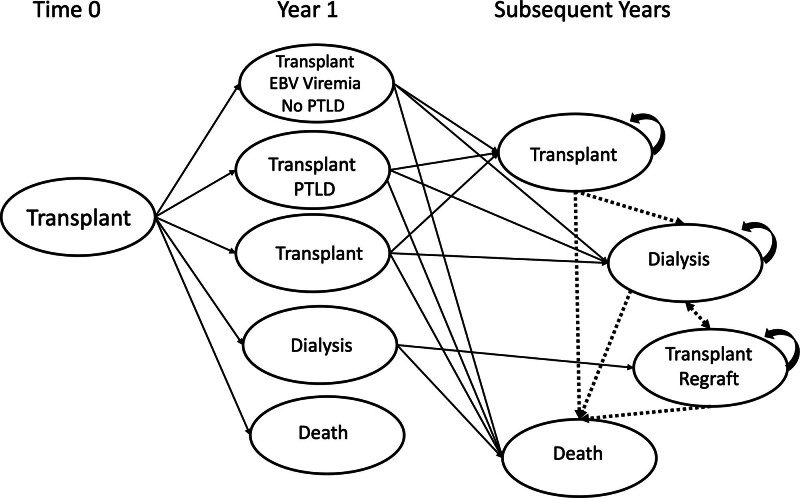
Markov model. PTLD, posttransplant lymphoproliferative disease.

Baseline probabilities are included in Table 1 and the model in Figure 1. The study assumed that there would be a 3% greater incidence of PTLD in high-risk compared with normal-risk recipients within the first year.^1,2^ The risks would not differ after the first year between cohorts.^1,2,4^ PTLD-associated mortality increased with age at diagnosis.^1^ After the first year, patients were either cured (with a functioning transplant or on dialysis) or died. There was no associated increase in graft loss with the development of PTLD.^1^ Patients with a failed transplant were able to be retransplanted and, presumably, were no longer EBV naive.

**TABLE 1.** Model probabilities and costs

Probabilities	Baseline (range)	References
Mortality		
Functioning transplant	Age adjusted	1,11
PTLD	Age adjusted (0.10–0.45)	
Dialysis after graft loss	Age adjusted	
Relative risk with rituximab use	1.0 (1.0–1.2)	
Graft loss		
Baseline	Age adjusted	1,11,12
Relative risk post–PTLD	1.0 (1.0–2.0)	
Incidence PTLD preemptive		
Age <18 y	0.05 (0.02–0.08)	1,2
Age ≥18 y	0.03 (0.01–0.06)	
EBV viremia post EBV D^+^R^–^	0.5 (0.4–0.8)	13, center experience
Relative risk of PTLD/viremia with intervention		
Rituximab	0.5 (0.25–0.75)	5,7-9,14-16
Antiviral	0.7 (0.60–0.90)	
Annual costs of transplant,$		
Year 1	156 032	11
Year ≥1	31 872	
Year of graft loss	145 123	
Reduced IS therapy, $		Center experience, 17
Viremia no PTLD	898 (750–1400)	
Dialysis	98 013 (80 632–109 090)	11
Screening		
Per test^*a*^	42.84 (30–80)	18
Total screening (and monitoring)	428 (360–1080)	
Intervention, $		
Antiviral		17
CMV D^–^R^–^	1530	
CMV D^+^R^–^	0	
CMV R^+^	765	
Rituximab		
Baseline dose (200 mg)	1712	
Low dose 100 mg	856	
High dose 375 mg/m^2^	5049	
Treatment PTLD,^*b*^ $	144 500 (80 000–200 000)	19
$ Constant US 2022		
Quality of life		
Transplant	0.82 (0.75–0.9)	20,21
Dialysis	0.71 (0.6–0.8)	
PTLD (year 1)	–0.25 (–0.4, –0.1)	
Discount rate	0.015 (0–0.5)	22
Time horizon	Lifetime (to age 95 y)	
Cycle	1 y	
Retransplantation		
Rate per 100 waitlist years	Age dependent	4,23

^*a*^Assume cost of EBV test similar to CMV.

^*b*^Difference in cost of functioning kidney transplant recipient patient with PTLD and without PTLD >1 y.

CMV, cytomegalovirus; EBV, Epstein-Barr virus; IS, immunosuppression; PTLD, posttransplant lymphoproliferative disease.

#### Objective 2: Quantify the Benefit of a Theoretical Prevention Strategy

Using the above-mentioned model, the high-risk cohort was compared with a similar high-risk cohort that had been given an intervention to reduce risk. The incidence of PTLD was reduced by the intervention over a range of risk ratios compared with the control nonintervention high-risk cohort.

##### Sensitivity Analysis

For both objectives 1 and 2, higher and lower rates of PTLD in high-risk compared with normal-risk groups were examined. The baseline model assumed there was no added risk of graft loss with PTLD, but rejection rates can be high with immunosuppression withdrawal.^24^ An earlier survey of pediatric centers estimated a hazard ratio of graft loss to be 1.59 at 5 y post PTLD.^12^ Therefore, increases in graft loss after PTLD up to 2-fold higher were examined. We assumed that no patient experienced graft failure and then developed PTLD within the first year.

### Secondary Analyses (Cost-effectiveness of Intervention)

Current practice: Monitor EBV viral load at 2 wk posttransplant in high-risk patients, monthly over the first 6 mo and then subsequently every 3 mo until 1 y. Those who develop a significant viral load have their immunosuppression reduced.^3^

#### Intervention 1: Rituximab Therapy

The first intervention compared current practice to low-dose rituximab (200 mg IV) at the time of transplantation in addition to standard immunosuppression therapy and viral load monitoring. Those who developed a significant viral load had their immunosuppression reduced.

The cost of the PTLD was considered to be a 1-time intervention.^19^ PTLD costs after the first year were not increased compared with non-PTLD transplant recipients. Costs were taken from the literature in 2022 US dollars using a consumer price index adjustment if required.^25^ Future costs were discounted. The perspective was from the federal government.

##### Sensitivity Analysis

Because the dose of rituximab is unclear, doses range from low (100 mg) to high (375/m^2^ or 600 mg). Higher doses may be associated with added morbidity and mortality but may be needed to be effective.^26^ The model included a potential increase in mortality with rituximab.

#### Intervention 2: Antiviral Therapy

The second intervention was oral valganciclovir daily for 6 mo. Viral load monitoring continued, immunosuppression was reduced if a significant viral load was detected within the first year. For the analysis, we assumed that all patients required this medication. However, CMV D^+^R^–^ would typically receive this medication for 6 mo, whereas CMV R^+^ would receive this medication for 3 mo and require only an additional 3 mo.^27^

TreeAge Pro Healthcare Version 2019 R2.1 (TreeAge Software, LLC, Williamstown, MA) was used to develop the Markov model and run the analyses (Figure 1). Patient and graft survival probabilities were taken from the literature as described in a previous publication to calculate life years.^11,23^ Life years were not discounted. QALYS were calculated using quality-of-life scaling factors for a functioning transplant, on dialysis, or with PTLD.^20,21^ QALYS were discounted.^22^ Costs were taken from published sources.^11,17-19^ One-way sensitivity analyses were run on all variables to see which were most important (key). Two-way analyses were conducted with key variables. In addition, microsimulations (1000 trials) were run to determine 95% confidence intervals (CIs) on the differences in effectiveness between the control and intervention cohorts. Log-normal distributions were applied to mortality, graft failure rate, and incidence of PTLD (first year) and normal distributions were applied to costs.^23^ The study used data from published reports and was deemed exempt from Institutional Review Board approval.

## RESULTS

### Main Analysis

Table 2 shows a range of cohorts by age at the time of transplant. As an example, a 40-y-old is projected to live 21.18 life years. If there is no PTLD, the recipient lives 21.37 y, but if PTLD develops in the first year, the projected remaining life years are only 15.03 y. Each high-risk 40-y-old EBV D^+^R^–^ recipient loses, on average, 0.192 life years or 0.134 QALYs. Slightly fewer life years are lost in younger recipients (age 10 y; 0.148 life years) and older recipients (age 60 y; 0.126 life years) due to lower case fatality rates in the young and higher competing risks of death in the old. In the sensitivity analysis, assuming PTLD was associated with a 2-fold increase in graft loss, a 40-y-old recipient experienced 0.138 fewer QALYs (compared with 0.134 QALYs assuming the baseline assumption of no increase in graft loss).

**TABLE 2.** Impact of PTLD by age: baseline analysis

Age, y	Life years with no PTLD	Life years with PTLD	Life years EBV D^+^R^–^ cohort	Loss of life years EBV D^+^R^–^ cohort (95% CI)	Loss of QALYS EBV D^+^R^–^ cohort (95% CI)
10	37.07	32.07	36.92	0.148 (0.146-0.150)	0.093 (0.091-0.095)
25	29.54	21.87	29.31	0.223 (0.220-0.226)	0.145 (0.142-0.148)
40	21.37	15.03	21.18	0.192 (0.189-0.193)	0.134 (0.131-0.137)
60	11.18	6.85	11.05	0.126 (0.124-0.128)	0.097 (0.095-0.099)

EBV, Epstein-Barr virus; PTLD, posttransplant lymphoproliferative disease; QALY, quality-adjusted life year.

The reduction in risk achieved by administering the intervention to the entire cohort, along with the incidence of PTLD in the first year, are the most important factors determining the benefit of the intervention. Assuming a reduction in PTLD of 50% with intervention and a baseline increase in absolute risk of PTLD of 3%, 0.067 QALYs could be gained in a 40-y-old recipient (Table 3). The intervention could add 0.134 QALYS if more effective or if the risk of PTLD was slightly higher.

**TABLE 3.** Incremental effectiveness: PTLD risk × intervention risk ratio: 40-y-old EBV D^+^R^–^ (QALYs, mean with 95% confidence intervals of the point estimate)

Added risk of PTLD	Risk ratio 0.75	Risk ratio 0.5	Risk ratio 0.25
2%	0.022 (0.020-0.024)	0.045 (0.042-0.048)	0.067 (0.065-0.069)
3%	0.033 (0.029-0.036)	0.067 (0.064-0.070)	0.100 (0.096-0.104)
4%	0.045 (0.042-0.048)	0.089 (0.086-0.092)	0.134 (0.131-0.137)

EBV, Epstein-Barr virus; PTLD, posttransplant lymphoproliferative disease; QALY, quality-adjusted life year.

### Secondary Analysis

**FIGURE 2.**
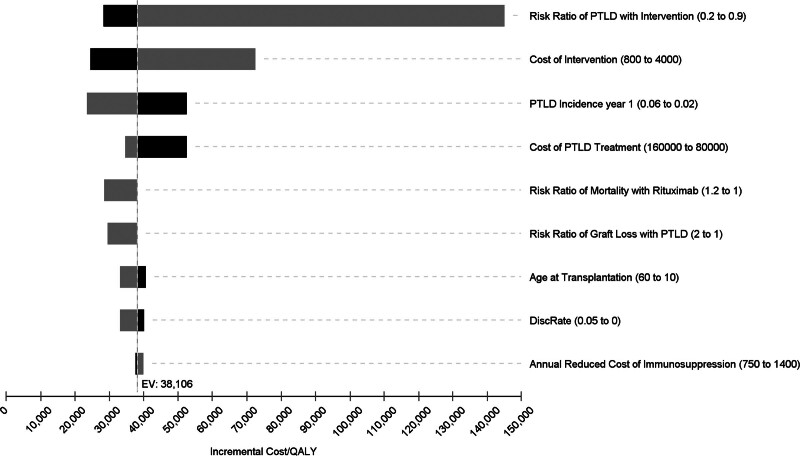
Incremental costs/QALY: intervention vs no intervention (40-y-old EBV D^+^R^–^). EBV, Epstein-Barr virus; PTLD, posttransplant lymphoproliferative disease; QALY, quality-adjusted life year.

Table 4 shows the incremental cost/QALY for the baseline assumptions by age. For example, in a 40-y-old, the incremental cost was $2537 and the incremental benefit was 0.067 QALYs ($38 106/QALY). Figure 2 shows the incremental cost/QALY over a range of several key variables (Tornado plot). The incremental cost/QALY was >$50 000 if the incidence rate of PTLD was <2% or the costs of the intervention (higher doses of rituximab) were >$3000.

**TABLE 4.** Incremental cost-effectiveness with baseline assumptions by age

Age, y	Incremental cost, US$	Incremental benefit, QALY	Incremental cost/QALY
10	1823 (1783-1863)	0.045 (0.043-0.047)	39 647 (36 347-42 947)
25	2794 (2744-2844)	0.072 (0.069-0.075)	38 886 (36 577-41 195)
40	2537 (2490-2584)	0.067 (0.064-0.071)	38 106 (37 354-38 858)
60	1632 (1587-1677)	0.048 (0.046-0.050)	33 814 (30 598-37 030)

QALY, quality-adjusted life year.

The added benefit of antiviral prophylaxis was 0.040 QALYs. The added costs would depend on whether the recipient was already on an antiviral based on their CMV status. Assuming the need for full therapy for 6 mo, the added cost was $2030 with an incremental cost/QALY of $51 011 compared with current practice. With the need for 3 mo of therapy, the incremental cost/QALY was $31 787. However, if the treatment only reduced PTLD by 10%, then the incremental cost/QALY rose to $131 026.

## DISCUSSION

PTLD is an important complication not only in kidney transplantation but also in non–renal solid organ transplantation and HCT.^1,7,28^ This study quantifies the loss of life years and QALYs from PTLD in EBV D^+^R^–^ kidney transplant recipients. Given a growing body of clinical observations, prevention of PTLD by using rituximab looks promising.^6-9,14-16^ This analysis suggests that efforts to reduce PTLD in high-risk kidney transplant recipients may be reasonably cost-effective. Unfortunately, it will be 4 y or more before we have an unbiased estimate of the benefits and safety of this approach (ClinicalTrials.gov ID NCT04989491). This trial is projected to be completed in late 2029. However, based on available data, this study estimates that 0.033–0.10 more QALYs can be gained with 25%–75% reductions in PTLD risk with intervention.

PTLD may be underreported in large databases.^4^ Our center has noted an increase in both the prevalence of R^–^ recipients and the rate of PTLD in these recipients despite viral screening and immunosuppression reduction (personal correspondence). Higher PTLD rates would support prevention and preemptive strategies. An increase in incidence within the first year from 3% to 4%, with a reduction in risk of 50% with intervention, would result in an increase in QALYs gained from 0.067 to 0.089. It is also unclear how well monitoring EBV viral load with preemptive immunosuppression lowering has helped. Studies suggest the correlation between EBV viral load and outcome is poor, and immunosuppression reduction comes with a risk of rejection.^13,24,29^

Rituximab appears to dramatically reduce the risk of PTLD in HCT. In one study, 12 of 147 untreated patients developed PTLD compared with none in 51 rituximab-treated patients.^6^ In a European pediatric center, prophylaxis reduced PTLD from 13.0% to 3.3% (hazard ratio, 0.23; *P* = 0.0045) in HCT recipients.^14^ In adult HCT recipients, Patel et al^15^ found that 6 of 43 untreated recipients developed PTLD compared with none in 43 rituximab-treated recipients. In the model, the baseline risk ratio was 0.5, and from the above, the risk reduction could be lower, favoring prophylaxis. However, the experience in HCT may not translate to the kidney transplant population and this is an important limitation of the secondary analysis.

The study incorporated a lower dose of rituximab at baseline. Studies in ABO incompatible kidney transplants have used both lower single doses (100–200 mg) and higher doses (375 mg/m^2^ for 1–4 doses) without any clinical differences in efficacy.^8,30-34^ Given that the infection is likely transmitted through passenger donor lymphocytes and the numbers would be small, a lower dose of rituximab may be sufficient. A dose-finding study would be the next step if the trial (ClinicalTrials.gov ID NCT04989491) demonstrates clinical benefit.

We also examined the use of valganciclovir. The benefit appears to be much smaller than that of rituximab, and it may be ineffective. In a systematic review, antivirals with activity against CMV trended toward reducing the risk of PTLD (risk ratio, 0.63; 95% CI, 0.17-2.37).^5^ In the model, we assumed a generous baseline risk ratio of 0.7 (95% CI, 0.6-0.9). Given the availability of antivirals and their use for CMV prophylaxis in many patients, this approach seems reasonable. However, the added burden of leukopenia may counteract any benefit.

To reduce PTLD risk, EBV D^–^ kidneys could be transplanted into EBV R^–^ recipients. The risk of PTLD in EBV D^–^R^–^ recipients is similar to D^+^/R^+^ and D^–^R^+^ recipients and much lower the risk in EBV D^+^R^–^ recipients.^35^ A serology matching strategy has been advocated for CMV in kidney transplantation.^36^ This study demonstrated that matching CMV D^–^R^–^ could increase patient survival and be cost-saving. This same would be for matching EBV D^–^R^–^. Our study would estimate that matching would result in 0.134 more QALYs at a savings of $1638 for the baseline 40-y-old recipient. However, <10% of donors are EBV D^–^ by serology, whereas 35%–40% of donors are CMV D^–^.^4^ Matching negative donors to negative recipients would be a greater challenge for EBV compared with CMV naive candidates. The negative impact of added wait time would need to be considered.

We considered an analysis of preemptive rituximab. Some adult and pediatric kidney transplant centers already administer the medication if the viral loads remain elevated despite immunosuppression reduction.^37,38^ This strategy would eliminate the need to treat most patients (reducing costs). However, it might not be as effective in reducing PTLD, and the required dose may be 4-fold higher (resulting in greater costs). Higher doses are also associated with morbidity and mortality in some studies, and the safety of preemptive and prevention strategies using rituximab must be examined in detail.^26^ The potential cost reduction relative to the reduced benefit is more complicated and deserves further study. The simplicity of prevention over preemptive therapy parallels the arguments for and against the approach of CMV disease prevention in kidney transplant practice.^27^

The study has several limitations. This study is a theoretical analysis based on historic published reports. It is highly likely that EBV D^+^R^–^ remains a significant health risk to recipients. Hopefully, better surveillance and PTLD treatment will reduce loss of life in the future. With respect to prevention, the most important variable determining cost-effectiveness is the efficacy of the intervention (see Figure 2). However, the cost-effectiveness of prevention may differ between privately insured and publicly managed healthcare systems. The costs of PTLD treatment and the costs of prevention are important yet opposing influences on overall cost-effectiveness. Higher PTLD treatment costs favor prevention, whereas higher prevention costs reduce the cost-effectiveness of prevention. There is likely some correlation between treatment costs and prevention costs (both may require rituximab). The study is not intended to substitute for clinical trials. The use of rituximab and valganciclovir described herein is currently off-label for the prevention of PTLD and requires further investigation before it can be broadly recommended. We did not specifically analyze live donor kidney transplant recipients but would not expect significant differences. The analysis assumed that PTLD occurred after the first year at rates that were similar between high-risk and normal-risk patients and that there would be no carry-over effect in the treated cohort. If this occurred, it benefited the intervention. The treated cohort might also benefit from a reduced rejection rate but may be subject to higher morbidity from infection.^26^

We incorporated a lower dose of rituximab, which would be relatively safe. We also assumed that patients with PTLD were not at higher risk of graft loss.^1^ This likely depends on center practice. Centers that completely withdraw immunosuppression could see higher risks of graft loss in those that develop PTLD.^12,24^ If this is the case, then our analysis underestimated the benefits of an effective prevention strategy. Given that some solid organ transplants have higher incidence rates of PTLD, a preventive strategy may be even more attractive in these populations.^28^

In summary, PTLD has a significant impact on survival in high-risk kidney transplant recipients. Preventive strategies may be cost-effective but would depend on the degree of effectiveness, safety, and cost.
